# Risk perception and priority setting for intervention among hepatitis C virus and environmental risks: a cross-sectional survey in the Cairo community

**DOI:** 10.1186/1471-2458-10-773

**Published:** 2010-12-20

**Authors:** Michaël Schwarzinger, Mostafa K Mohamed, Rita R Gad, Sahar Dewedar, Arnaud Fontanet, Fabrice Carrat, Stéphane Luchini

**Affiliations:** 1 INSERM UMR_S 707, and UPMC, Univ Paris 06, F-75012, Paris, France; 2 Center for Health Policy, Freeman Spogli Institute for International Studies/Center for Primary Care & Outcomes Research, School of Medicine, Stanford University, CA 94305-6019, USA; 3 Department of Community, Environmental and Occupational Medicine, Faculty of Medicine, Ain Shams University, Cairo, Egypt; 4 Emerging Disease Epidemiology Unit, Institut Pasteur, F-75015, Paris, France; 5 AP-HP, Hôpital Saint-Antoine, Unité de Santé Publique, F-75012, Paris, France; 6 GREQAM UMR 6579-IDEP, CNRS, Centre de la Vieille Charité, F-13002, Marseille, France

## Abstract

**Background:**

Hepatitis C virus (HCV) recently emerged as a major public health hazard in Egypt. However, dramatic healthcare budget constraints limit access to the costly treatment. We assessed risk perception and priority setting for intervention among HCV, unsafe water, and outdoor air pollution in Cairo city.

**Methods:**

A survey was conducted in the homes of a representative sample of household heads in Cairo city. Risk perception was assessed using the "psychometric paradigm" where health hazards are evaluated according to several attributes and then summarized by principal component analysis. Priority setting was assessed by individual ranking of interventions reducing health hazards by 50% over five years. The Condorcet method was used to aggregate individual rankings of the three interventions (main study) or two of three interventions (validation study). Explanatory factors of priority setting were explored in multivariate generalized logistic models.

**Results:**

HCV was perceived as having the most severe consequences in terms of illness and out-of-pocket costs, while outdoor air pollution was perceived as the most uncontrollable risk. In the main study (n = 2,603), improved water supply received higher priority than both improved outdoor air quality (60.1%, *P *< .0001) and screening and treatment of chronic hepatitis C (66.3%, *P *< .0001), as confirmed in the validation study (n = 1,019). Higher education, report of HCV-related diseases in the household, and perception of HCV as the most severe risk were significantly associated to setting HCV treatment as the first priority.

**Conclusions:**

The Cairo community prefers to further improving water supply as compared to improved outdoor air quality and screening and treatment of chronic hepatitis C.

## Background

Decision-makers at all levels of the healthcare system should incorporate both scientific evidence and public values in setting priorities [1]. Efforts to improve priority setting in developing countries have focused on providing accurate information and tools such as the Burden of Disease study [2-5], National Health Accounts [6,7], and cost-effectiveness analysis of health interventions [8-10]. Priority setting remains however a value laden and political process [11-13]. Households of developing countries shoulder most of the burden of health financing [14]. Accordingly, their demand for publicly-subsidized health interventions should be better taken into account [15,16].

In Egypt, 62% of total healthcare expenditures were in the form of out-of-pocket payments in 2002 [17]. In addition, financial viability of environmental investments made by donors eventually relies on households, e.g., water tariffs were increased to cover the costs of maintenance of improved water supply in Cairo city [18]. Previous comparative risk assessments showed that air pollution ranked as a higher risk than unsafe water, sanitation and hygiene [19,20]. Meanwhile, screening and treatment of chronic hepatitis C emerged as a public health priority to reduce both the burden of liver disease and the transmission of hepatitis C virus (HCV).

About 15% of 59 million Egyptians in 1996 were estimated to test positive for anti-HCV antibody [21]. About 60% of those have a chronic hepatitis C with positive HCV-RNA [22], but most of them remain unaware of their diagnosis due to a silent infection and out-of-pocket costs of laboratory testing [22,23]. While the combination of pegylated interferon alpha and ribavirin is recommended for the treatment of chronic hepatitis C in high-income countries [24], the combination therapy showed sustained viral response rates exceeding 60% in Egyptian patients who are mostly infected by the genotype 4 [25,26]. Recently, the Egyptian National Control Strategy for Viral Hepatitis estimated that 2% of 600,000 Egyptians needing treatment were actually treated and targeted treatment for 20% by 2012 under publicly-subsidized schemes [27].

While the combination therapy is not affordable for most Egyptian patients (about 3,000 euros for a full 48 week course of treatment) [27], the public value attached to screening and treatment of chronic hepatitis C in Egypt is unknown. We conducted a cross-sectional survey among a large representative sample of household heads in Cairo city to assess the demand for screening and treatment of chronic hepatitis C as compared to improved water supply and improved outdoor air quality. In this paper, we report on risk perception and the priority set by a voting procedure among these health interventions competing for scarce healthcare resources.

## Methods

### Study overview

Face-to-face interviews were conducted in the homes of a random sample of household heads in Cairo city from October 2004 to July 2005. Each household head was randomly assigned to one out of 25 types of questionnaire depending on the number and order of health hazards presented, and the format used to elicit household willingness to pay for interventions [28-30]. In this paper, we report on risk perception (section 1 of the questionnaire) and the priority set (section 2 of the questionnaire) by participants presented with at least two health hazards. Participants presented with all three health hazards and having set priority for intervention were identified as the "main study". Participants presented with two health hazards were identified as the "validation study" to test the reliability of priority setting from the main study. Participants presented with only one health hazard were discarded from this analysis on priority setting. Report of the willingness to pay results according to the elicitation format (section 3 of the questionnaire) is provided elsewhere. The study was approved by the institutional review board at the University of Ain Shams, Cairo, and informed consent was obtained from each participant.

### Selection of participants

Based on the 1996 Cairo census, 1,677,981 households consisting of 6,789,479 individuals were distributed among 32 geographic areas and divided into 100 clusters of similar size in Cairo city. A stratified sample of households was selected where strata were geographic areas. Twenty-five Egyptian interviewers were trained in a pilot study and supervised during the survey. Door-to-door recruitment started from the right-hand street of the principal underground station of each cluster until 47 (0.28%) household heads were interviewed. The interviewers came late purposely with one-third interviews starting after 6:45 pm; when household head could not be found, interviewers knocked at the next neighboring door. Interviewers completed consecutively numbered booklets of 30 questionnaires, while the type of questionnaire was randomly allocated per booklet.

### Questionnaire

The Arabic questionnaire included four sections. In the first section, risk perception was assessed using the "psychometric paradigm" where health hazards are evaluated according to several attributes and then summarized by principal component analysis [31]. In the second section, priority setting among health hazards was based on presentation of the following hypothetical interventions: the provision of screening and treatment of adults with chronic hepatitis C, a process of purification of water, and a process of waste management to avoid open-air waste burning. Each intervention aimed to reduce overall risk by 50% over five years depending on household monthly payments to a not-for-profit company. Expected benefits for the household were detailed for each intervention as well as potential number of cases avoided in Cairo city by means of visual aids [32]. Participants were asked to rank presented interventions from first to last priority for intervention to be addressed by the not-for-profit company. In the third section, the participant was asked to provide his/her maximum willingness to pay for interventions according to the elicitation format randomly allocated. In the last section, socio-demographic variables and the relative severity of one's health status were recorded.

### Variables

Risk perception was assessed by eight 10-point scales: the difficulty to avoid health hazards; the consequences of health hazards in terms of illness severity, out-of-pocket costs, work absenteeism; and the value of each hypothetical intervention. Consequences of health hazards were assessed both in the short run, i.e., directly after exposure, and in the long run, i.e., years after exposure. The best organization to provide each intervention was also assessed by a categorical variable. A continuous household income variable was estimated using an interval regression where the dependent variable was the response given among 8 income categories and explanatory variables were socio-economic variables as well as ownership of washing machine, dishwasher, air conditioning, and private car [33]. A 100-point visual analogue scale (VAS) was used to assess the relative severity of one's health status during the last month [23]. The date of interview was matched to the latest measurement of air quality in Cairo city, i.e., monthly mean of suspended particles with diameter less than 10 micrometer (PM_10_), and 24 h mean of Sulphur Dioxide (SO_2_) [34].

### Statistical methods

Many of the attributes of risk perception were correlated with each other. A principal component analysis of qualitative data was carried out on pooled health hazards (n = 9,847) with optimal monotonic transformation of 10-point scales [35]. Principal components were retained on the basis of the eigenvalues-greater-than-one rule, and standardized scores of principal components were used subsequently in regression analysis on individual data.

The Condorcet method was used to aggregate the individual rankings of interventions [36]. The Condorcet method yields the "best compromise" intervention, the one that the majority will find to be the least disagreeable, even if not their preferred intervention. The count is conducted by pitting each intervention against every other in a series of pairwise comparisons. The winner of each pairing is the intervention preferred by a majority of participants. Binomial proportions were tested against the hypothesis that the proportion is 50%. In the main study where participants ranked the three interventions, a poll of 2,645 participants allowed a maximum margin of sampling error of 1.9% for an observed percentage of 50%. In the validation study where participants were presented with only two out of three interventions, a poll of 345 participants allowed a maximum margin of sampling error of 5.3% for each pairwise comparison.

Explanatory factors of priority setting in the main study were selected by a backward procedure at the 0.05 level in generalized logistic models adjusted for interviewer and stratified for geographic area with finite population correction included in the variance estimation. All analyses were based on two-sided *P *values, with *P *< .05 considered to indicate statistical significance. All analyses were performed on SAS 9.1.3 statistical software (SAS Institute, Cary, NC).

## Results

### Characteristics of households surveyed

Overall 3,702 household heads from Cairo city were interviewed to assess risk perception and priority setting for intervention among HCV, unsafe water, and outdoor air pollution. In the main study, 58 (2.2%) of 2,661 participants did not rank the three interventions presented and were excluded. In the validation study, 22 (2.1%) of 1,041 participants did not rank the two interventions presented and were excluded. As shown in Table 1 the 3,622 respondents were 83.6% male and had a mean (SD) age of 49.8 (12.2) years, 32.6% obtained a university degree and 67.5% had a job. Respondents had a mean (SD) health status score of 76.7 (16.9) on the VAS. The mean (SD) household income was 91 (64) per month in 2005 US dollars. About 7% of households reported diseases related to HCV as compared to 20.7% for unsafe water (*P *< .0001) and 36.2% for outdoor air pollution (*P *< .0001). Monthly mean (SD) PM_10 _was 239 (62) μg/m^3 ^at time of interview. Respondents in the validation study did not differ from those in the main study.

**Table 1:** Characteristics of households surveyed in Cairo city (n = 3,622)

	Overall (n = 3,622)	Main study with 3 health hazards (n = 2,603)	Validation study with 2 health hazards (n = 1,019)
**Characteristics of household heads**			
Male, No. (%)	3,029 (83.6)	2,184 (83.9)	845 (82.9)
Age, mean (SD), yr	49.8 (12.2)	49.7 (12.2)	50.1 (12.2)
Education, No. (%)			
Primary school	986 (27.2)	717 (27.6)	269 (26.4)
Secondary school	1,455 (40.2)	1,041 (40.0)	414 (40.6)
University	1,181 (32.6)	845 (32.4)	336 (33.0)
Main occupation, No. (%)			
Public sector employee	930 (25.7)	669 (25.7)	261 (25.6)
Private sector employee	897 (24.7)	639 (24.5)	258 (25.3)
Own business	619 (17.1)	444 (17.1)	175 (17.2)
Retired/housewife	1,176 (32.5)	851 (32.7)	325 (31.9)
Health status, mean (SD), VAS score	76.7 (16.9)	76.5 (16.8)	77.2 (17.3)
**Characteristics of households**			
Number of adults, mean (SD)	3.0 (1.5)	3.0 (1.5)	2.9 (1.5)
Number of children, mean (SD)	1.0 (1.2)	1.0 (1.2)	1.0 (1.3)
Monthly income, mean (SD), EGP	521 (369)	520 (364)	522 (383)
New rental, No. (%)	443 (12.2)	327 (12.6)	116 (11.4)
Bimonthly water bill, mean (SD), EGP	12.8 (9.7)	12.9 (9.8)	12.8 (9.6)
Diseases related to health hazards, No. (%)*			
Hepatitis C virus	238 (7.2)	189 (7.3)	49 (7.2)
Unsafe water	681 (20.7)	528 (20.3)	153 (22.5)
Outdoor air pollution	1,186 (36.2)	948 (36.4)	238 (35.3)
**Air quality at time of interview**			
Monthly Particulate Matter PM10, mean (SD), μg/m^3^	239 (62)	239 (62)	239 (64)
24 h SO_2_, mean (SD), μg/m^3^	30.1 (12.2)	31.1 (12.5)	29.9 (11.4)

* Self-declared diseases in the household included overall 5.7% chronic hepatitis, 1.7% liver failure, 1.1% liver cancer related to hepatitis C virus; 17.6% diarrhea, 2.5% acute hepatitis A, 0.9% typhoid, 1.6% kidney failure related to unsafe water; 24.5% asthma attacks, 23.0% chronic bronchitis, 3.8% heart diseases, 1.4% lung cancer related to outdoor air pollution

Note: Except where stated otherwise, values are expressed in percentage of subjects.

Abbreviations: VAS, Visual Analogue Scale; EGP, Egyptian Pound (2005 US$ 1 = EGP 5.75)

### Risk perception

Three principal components of risk perception were retained which together accounted for 68% of the variance (Table 2). The first and second components were labeled "severe risk" with high correlation to the severity of consequences in the long run (35.9%) and the short run (19.7%), respectively. The third component (12.9%) was associated with difficulty to avoid health hazard, undervaluation of intervention, and mistrust in the Ministry of Health and Population to provide intervention. This component was labeled "uncontrollable risk". HCV was perceived as the most severe risk with higher mean standardized scores on the first and second principal components, while outdoor air pollution was perceived as the most uncontrollable risk.

**Table 2:** Principal component analysis and standardized scores of health hazards (n = 9,847).

Attributes* and loadings after varimax rotation	Factor 1	Factor 2	Factor 3
	"Severe risk in the long run"	"Severe risk in the short run"	"Uncontrollable risk"
Out-of-pocket costs in the long run	0.91	0.14	-0.01
Work absenteeism in the long run	0.90	0.13	-0.02
Severity in the long run	0.90	0.15	0.02
Out-of-pocket costs in the short run	0.13	0.91	0.02
Severity in the short run	0.13	0.88	0.05
Work absenteeism in the short run	0.14	0.86	0.05
Difficulty to avoid health hazard	0.05	0.08	0.75
Undervaluation of intervention reducing health hazard by 50%	-0.16	-0.04	0.71
Mistrust in MOHP to provide intervention	0.04	0.03	0.35
**Variance explained by each factor, %**	35.9	19.7	12.9
**Standardized scores on principal components, mean (SD)**			
Hepatitis C virus (n = 3,277)	0.090 (0.897)	0.358 (1.063)	-0.173 (1.031)
Outdoor air pollution (n = 3,286)	0.055 (0.964)	0.033 (0.937)	0.368 (0.804)
Unsafe water (n = 3,284)	-0.145 (1.111)	-0.392 (0.841)	-0.194 (1.042)

* Attributes are coded so that high value pertains to greater risk severity.

Abbreviations: MOHP: Egyptian Ministry of Health and Population

### Priority setting using a voting procedure

Improved water supply, improved outdoor air quality, and screening and treatment of chronic hepatitis C were ranked first priority by 46.1%, 27.6%, and 26.3% household heads in the main study, respectively (Table 3). According to the Condorcet method, the majority of respondents preferred improved water supply to both screening and treatment of chronic hepatitis C (66.3%, *P *< .0001) and improved outdoor air quality (60.1%, *P *< .0001), while improved outdoor air quality was preferred to screening and treatment of chronic hepatitis C (55.9%, *P *< .0001). Priorities set in the main study were confirmed in the validation study where respondents were presented with only two out of three health hazards, although pairwise comparison of outdoor air pollution to HCV did not reach statistical significance.

**Table 3:** Priority setting for intervention among hepatitis C virus, unsafe water, and outdoor air pollution (n = 3,622)

Pairwise comparisons of interventions in the Condorcet method	Main study where respondents ranked three health hazards				Validation study where respondents ranked two health hazards			
	n	%	95% CI	P Value	n	%	95% CI	P Value
Improved water supply preferred to screening and treatment of chronic hepatitis C	2,603	66.3	64.4 to 68.1	< .0001	345	62.0	56.7 to 67.2	< .0001
Improved water supply preferred to improved outdoor air quality	2,603	60.1	58.2 to 62.0	< .0001	336	58.6	53.2 to 64.0	< .001
Improved outdoor air quality preferred to screening and treatment of chronic hepatitis C	2,603	55.9	53.9 to 57.8	< .0001	338	52.4	46.9 to 57.8	.38

Note: Binomial proportions were tested against the hypothesis that the proportion is 50%.

Abbreviations: 95% CI, 95% confidence interval.

### Explanatory factors of priority setting

Factors that may change significantly the first priority set for intervention were selected in multivariate analysis (Table 4). Setting HCV and outdoor air pollution as the first priority for intervention over unsafe water was associated with higher education (*P *< .0001) and the perception of unsafe water as a controllable risk (*P *< .05). Setting HCV as the first priority for intervention was also associated with the presence of HCV-related diseases in the household (*P *< .01) and the perception of HCV as a severe risk in the short run (*P *< .0001), while setting outdoor air pollution as the first priority for intervention was associated with the presence of air pollution-related diseases in the household (*P *< .0001), the perception of air pollution as a severe risk in the short run (*P *< .01), and the perception of air pollution as a controllable risk (*P *< .01).

**Table 4:** Odds ratio for setting hepatitis C virus or outdoor air pollution first priority over unsafe water in the main study (n = 2,603)

	Unadjusted odds ratio (95%CI)			Adjusted odds ratio (95%CI)		
	Hepatitis C virus	Outdoor air pollution	p-value	Hepatitis C virus	Outdoor air pollution	p-value
**Characteristics of household heads**						
			
Gender, female vs. male	0.92 (0.72 to 1.19)	0.85 (0.65 to 1.11)	.48			
			
Age (yr), older (> 55) vs. younger (< 44)	0.93 (0.70 to 1.23)	0.97 (0.80 to 1.87)	.97			
Age (yr), median (45 to 54) vs. younger (< 44)	0.95 (0.73 to 1.23)	1.02 (0.82 to 1.28)				

Education, university vs. primary school	1.85 (1.26 to 2.71)	1.56 (1.16 to 2.10)	< .0001	1.91 (1.30 to 2.81)	1.57 (1.17 to 2.10)	< .0001
Education, secondary school vs. primary school	1.51 (1.17 to 1.95)	1.04 (0.78 to 1.37)		1.45 (1.13 to 1.87)	1.04 (0.80 to 1.38)	

Private sector employee vs. Public sector employee	0.89 (0.69 to 1.16)	1.02 (0.79 to 1.33)	.69			
Own business vs. Public sector employee	0.70 (0.47 to 1.05)	0.86 (0.63 to 1.17)				
Retired/housewife vs. Public sector employee	0.83 (0.61 to 1.14)	0.88 (0.67 to 1.15)				
			
Health status (VAS), high (> 85) vs. low (<= 70)	1.33 (0.94 to 1.89)	1.01 (0.74 to 1.38)	.02			
Health status (VAS), median (71 to 85) vs. low (<= 70)	0.93 (0.69 to 1.24)	0.67 (0.50 yo 0.91)				
			
**Characteristics of households**						
			
Number of adults, more than two vs. less	0.85 (0.69 to 1.04)	0.88 (0.74 to 1.06)	.19			
			
Number of children, at least one child vs. none	0.82 (0.64 to 1.05)	0.98 (0.82 to 1.17)	.27			
			
Monthly income (EGP), high (> 494) vs. low (< 354)	1.49 (1.04 to 2.14)	1.15 (0.83 to 1.58)	.14			
Monthly income (EGP), median (355 to 494) vs. low (< 354)	1.07 (0.80 to 1.42)	1.13 (0.89 to 1.42)				
			
New rental, no vs. yes	1.18 (0.85 to 1.63)	0.99 (0.72 to 1.35)	.54			
			
Bimonthly water bill (EGP), high (> 10) vs. low (< 10)	1.36 (1.01 to 1.83)	1.09 (0.86 to 1.37)	.13			
			
**Perception of hepatitis C virus hazard**						

Diseases related to health hazard in household, yes vs. no	1.92 (1.34 to 2.74)	0.99 (0.70 to 1.41)	< .001	1.78 (1.23 to 2.58)	0.96 (0.66 to 1.38)	< .01

Severe risk in the long term, yes vs. no	0.99 (0.66 to 1.47)	0.88 (0.65 to 1.19)	.68			

Severe risk in the short term, yes vs. no	2.16 (1.64 to 2.84)	1.31 (0.97 to 1.77)	< .0001	2.22 (1.69 to 2.91)	1.04 (0.76 to 1.43)	< .0001

Uncontrollable risk, yes vs. no	0.90 (0.69 to 1.18)	0.68 (0.52 to 0.89)	.02			
			
**Perception of outdoor air pollution hazard**						

Diseases related to health hazard in household, yes vs. no	1.43 (1.08 to 1.89)	2.02 (1.59 to 2.57)	< .0001	1.40 (1.06 to 1.84)	1.99 (1.57 to 2.54)	< .0001

Severe risk in the long term, yes vs. no	0.83 (0.58 to 1.19)	0.97 (0.72 to 1.30)	.56			

Severe risk in the short term, yes vs. no	1.16 (0.89 to 1.52)	1.53 (1.15 to 2.03)	.02	0.81 (0.62 to 1.06)	1.44 (1.05 to 1.95)	< .01

Uncontrollable risk, yes vs. no	0.58 (0.43 to 0.76)	0.50 (0.36 to 0.70)	< .0001	0.71 (0.54 to 0.95)	0.56 (0.41 to 0.77)	< .01

**Perception of unsafe water hazard**						
			
Diseases related to health hazard in household, yes vs. no	1.27 (0.89 to 1.81)	1.26 (1.00 to 1.58)	.12			
			
Severe risk in the long term, yes vs. no	1.03 (0.72 to 1.47)	1.05 (0.80 to 1.38)	.94			
			
Severe risk in the short term, yes vs. no	1.14 (0.80 to 1.64)	1.34 (1.00 to 1.77)	.13			

Uncontrollable risk, yes vs. no	0.71 (0.54 to 0.91)	0.66 (0.52 to 0.85)	< .01	0.79 (0.61 to 1.03)	0.78 (0.63 to 0.97)	.05

Note: All generalized logistic models were adjusted for interviewer and stratified for geographic area with finite population correction included in the variance estimation.

Note: We estimated multivariate odds ratios after backward stepwise selection, with P < .05 used as the cutoff for retention in the model. Standardized scores of principal components were dichotomized (Yes for score above 0, No otherwise).

Abbreviations: HCV, hepatitis C virus; VAS, Visual Analogue Scale; EGP, Egyptian Pound; 95%CI, 95% confidence interval

**Figure 1:**
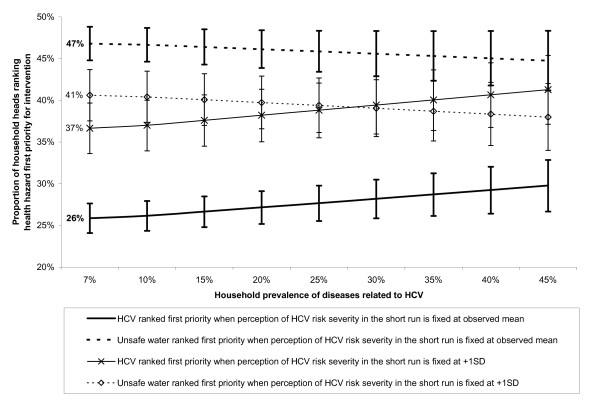
**Sensitivity analysis on priority setting for intervention by prevalence of HCV in the household and perception of HCV severity in the short run**. The Figure shows the predicted proportion of household heads ranking HCV and unsafe water as the first priority for intervention with variation of the household prevalence of diseases related to HCV (from 7% to 45%),[21] and the perception of HCV severity in the short run (fixed at observed mean or +1 Standard Deviation). All other explanatory factors of priority setting selected in the multivariate model (Table 4) were fixed at mean observed values (n = 2,603). The proportion of household heads ranking improved outdoor air quality as the first priority is complementary and it is not shown. Vertical bars show 95% confidence interval of the proportion.

We performed a sensitivity analysis to test whether screening and treatment of chronic hepatitis C might be set as the first priority at the population level following increasing reports of HCV-related diseases in the household or a worsening perception of HCV as a severe risk in the short run, all other things being equal. As shown in Figure 1 increasing reports of HCV-related diseases in the household were unlikely to change priority setting unless the perception of HCV as a severe risk in the short run worsens simultaneously to dramatic ends.

## Discussion

### Main results

In the main study, the majority of 2,603 representative household heads in Cairo city set higher priority for improved water supply as compared to both screening and treatment of chronic hepatitis C (66.3%, *P *< .0001) and improved outdoor air quality (60.1%, *P *< .0001). Improved water supply was also set as the first priority by the majority in the validation study where respondents were presented with only two out of three interventions. While HCV was perceived as having the most severe consequences in terms of illness and out-of-pocket costs in comparison to unsafe water and outdoor air pollution, screening and treatment of chronic hepatitis C was unlikely to be set as the first priority unless reports of HCV-related diseases and the perception of HCV as a severe risk increase to dramatic ends.

### Explanation for the findings

To our knowledge, the present study is the first opinion poll conducted among a large representative sample in a developing country to measure community ranking of interventions reducing major health hazards. Based on household reports, our selected health hazards had dramatically manifested into related diseases, i.e., 7.2% for HCV (including 5.7% chronic hepatitis C), 20.7% for waterborne diseases (including 17.6% diarrhea), and 36.2% for outdoor air pollution (including 24.5% asthma attacks). Our disease assessment was limited to self-declaration, but the rates seem reasonable when compared to previous surveys conducted in Cairo city; in 1996, 11.0% of 1,603 individuals tested positive for anti-HCV antibody [21], and in 2005, 15.6% and 18.4% of 218 mothers reported episodes of diarrhea and cough among their children under five years old, respectively [37].

We assessed risk perception using the "psychometric paradigm". Our study differs from the classical approach in several aspects [31]. We focused on three health hazards instead of assessing dozens of health hazards, and we selected risk attributes accordingly (e.g., "voluntariness" was not assessed). We also added diseases related to health hazards in the assessment of risk perception to aid interviewees in the understanding of health hazards. However, the preeminence of risk perception along severity of consequences (56% of variance) replicates previous findings in both developed and developing countries about the so-called "dreadfulness" of risk [31,38,39]. In our study, HCV was perceived as more severe than environmental risks. By analogy, Bronfman et al found that HIV was perceived as more "dreadful" than environmental risks in Chile [38]. The air quality in Cairo at time of interview reached three times the WHO target for monthly PM_10 _set at 70 μg/m^3 ^[40]. Presumably, the very bad air quality explains the perceived lower controllability of outdoor air pollution as compared to HCV and unsafe water.

The main study and the validation study showed consistently that improved water supply received the highest priority in spending additional public health funds. It conveys the primary concern of the poor population for an improved access to safer public water as shown by the strong association of priority setting with education (*P *< .0001). All other things being equal, increasing reports of HCV-related diseases in the household are unlikely to alter that improved water supply should be addressed before screening and treatment of chronic hepatitis C.

### Study limitations

The study results are limited to our selection of public health hazards and interventions, as well as the Cairo population surveyed. The validation study presenting only two out of three health hazards provided support to the priority set in the main study presenting all three health hazards. However, the inclusion of other health hazards, e.g., child undernutrition [10], may have led to other priority setting. We chose a similar relative risk reduction of 50% to facilitate understanding and comparison of interventions. This figure is realistic since unsafe water and outdoor air pollution hazards could be brought to acceptable levels, and pegylated interferon alpha and ribavirin combination therapy showed sustained viral response rates exceeding 60% in Egypt [25,26]. While the addition of new protease inhibitors to the combination therapy may achieve higher sustained viral response rates [41-43] and the case for priority setting would become even more critical due to increasing drug costs, we can only assume that our results would generalize to higher levels of relative risk reduction.

Community interventions were also selected to be neutral to individual behavior and income, e.g., we discarded road traffic interventions. However, respondents perceived that outdoor air pollution was more "uncontrollable". In particular, the process of waste management to avoid open-air waste burning had a significantly lower value than screening and treatment of adults chronically infected by HCV (*P *< .0001). It may relate to the knowledge of other major sources of outdoor air pollution with lower controllability including road traffic, industries, and sand storms [40], while the very bad air quality at time of interview could result in the mistrust in any outdoor air pollution intervention. Alternatively, one could hypothesize that Egyptians feel less at risk of contracting HCV because awareness campaigns enhanced knowledge of modes of transmission and methods of prevention among the general population [27]. Finally, a random sample of 3,622 household heads completed the Cairo survey. All socio-demographic variables were similar between the main and validation studies supporting the selection of a representative sample of the Cairo community.

### Implications for health decision makers

Perception of risks proved to differ between experts relying more on technical estimates of annual fatalities and laypeople who rely more on other hazard characteristics such as "dread" [31]. Considerable efforts have been done to provide evidence-based health risk assessment based on Disability-Adjusted Life Years (DALYs) lost at the global and regional level [3,4,10,44]. This information is useful to raise awareness, and set the health policy agenda. Whether this information reflects community values at the country level remains debatable [45,46], and our study allows comparison of available information for health decision making to community values in Egypt.

According to the 2002 Egyptian Burden of Disease study provided by WHO, 13.6 million DALYs were lost. Unsafe water accounted for 464,000 (3.39%) DALYs lost due to diarrheal diseases [3]. Outdoor air pollution accounted for 154,700 (1.13%) DALYs lost [47]. HCV accounted for 134,000 (0.98%) to 221,000 (1.62%) DALYs lost (i.e., categories "hepatitis C" added to a proportion assumed by the authors of 40% to 70% of "cirrhosis" and "liver cancer" attributable to HCV). In comparison to priorities implied by the magnitude of DALYs lost in Egypt, targeting unsafe water hazard was ranked similarly the highest priority by the Cairo community. Assumingly, the primary concern for an improved water supply in Cairo city should generalize to Egypt of lower education on average. While the community ranked improved outdoor air quality higher priority than screening and treatment of chronic hepatitis C, comparison to priorities implied by DALYs lost is difficult due to DALY estimate uncertainties [5,48], as well as the particular epidemiological situation of Cairo city with regard to both health hazards.

While our study results are in agreement with the expert-based health risk assessment above, our survey goes further by shedding light on the political implications of priority setting among additional publicly-subsidized health interventions. First, previous qualitative surveys showed consistently that health decision makers thought that a participatory process ensuring equal participation of all stakeholders was a necessary condition to fair priority setting [11-13]. Among stakeholders, the households' demand for publicly-subsidized health interventions seems difficult to ignore in developing countries. We found that an opinion poll was indeed feasible with about 98% of the 3,702 household heads volunteering to set such priorities. In addition, the costs of conducting an opinion poll are negligible as compared to the costs of health interventions.

Second, priority setting was strongly associated with risk perceptions in the community. Quite logically, respondents gave a higher priority to target HCV or outdoor air pollution when either health hazard was perceived as more dreadful and costly, or had even manifested into diseases in their household. Priority setting was also associated with the perception of "controllability" over environmental risks. When environmental risks were deemed uncontrollable, respondents gave a higher priority to target water supply. When environmental risks were perceived as controllable, respondents gave a higher priority to target outdoor air pollution and, to a lesser extent, HCV.

Finally, setting HCV treatment as the first priority was strongly associated with higher education. Accordingly, targeting treatment of 20% of 600,000 Egyptians with chronic hepatitis C by 2012 raises some ethical concerns. In the absence of a national HCV screening program to detect asymptomatic individuals with chronic hepatitis C [27], candidates for treatment are self-selected on their awareness of HCV and affordability of laboratory testing. This makes the more educated and wealthy people more likely candidates for treatment, thus targeting treatment at the population level reflects mostly their priority, and, in turn, the poor may lose out to the rich under publicly-subsidized schemes.

## Conclusions

While screening and treatment of chronic hepatitis C emerged as a public health priority in Egypt, we found that the Cairo community attached more value to improving water supply. We believe such information on public values is invaluable in the process of a fair priority setting for health interventions [49,50]. We encourage future use of the methods presented here in other countries, e.g., in sub-Saharan Africa where significant resources have been allocated to HIV/AIDS as compared to other life-threatening diseases [49].

## Competing interests

The authors declare that they have no competing interests.

## Authors' contributions

MS originated the study, assisted with the study design and implementation, completed the analyses, and led the writing. MKM supervised all aspects of the study implementation. RRG and SD assisted with the study implementation and analyses. AF and FC assisted with analyses. SL designed the study and assisted with analyses. All authors helped to conceptualize ideas, interpret findings, and review drafts of the article.

## Pre-publication history

The pre-publication history for this paper can be accessed here:

http://www.biomedcentral.com/1471-2458/10/773/prepub
